# Anti-gastric cancer activity in three-dimensional tumor spheroids of bufadienolides

**DOI:** 10.1038/srep24772

**Published:** 2016-04-21

**Authors:** Jixia Wang, Xiuli Zhang, Xiaolong Li, Yun Zhang, Tao Hou, Lai Wei, Lala Qu, Liying Shi, Yanfang Liu, Lijuan Zou, Xinmiao Liang

**Affiliations:** 1 Key Lab of Separation Science for Analytical Chemistry, Dalian Institute of Chemical Physics, Chinese Academy of Sciences, Dalian, China; 2 Department of Radiation Oncology, Second Affiliated Hospital of Dalian Medical University, Dalian 116027, China; 3 Co-innovation Center of Neuroregeneration, Nantong University, Nantong, 226019, China

## Abstract

Multicellular spheroids of cancer cells have been increasingly used to screen anti-tumor compounds, owing to their *in vivo* like microenvironment and structure as well as compatibility to high-throughput/high-content screening. Here we report the potency and efficacy of a family of bufadienolides to inhibit the growth of gastric cancer cell line HGC-27 in three-dimensional (3D) spheroidal models. Examining the morphological and growth patterns of several cell lines in round-bottomed ultra-low attachment microplate suggested that HGC-27 cells formed reproducibly multicellular spheroidal structures. Profiling of 15 natural bufadienolides isolated from toad skin indicated that 8 14-hydroxy bufadienolides displayed inhibitory activity of the growth of HGC-27 spheroids in a dose-dependent manner. Notably, compared to clinical drugs taxol and epirubicin, active bufadienolides were found to penetrate more effectively into the HGC-27 spheroids, but with a narrower effective concentration range and a shorter lasting inhibitory effect. Furthermore, compared to two-dimensional (2D) cell monolayer assays, active bufadienolides exhibited weaker efficacy and different potency in 3D spheroid model, demonstrating the great potential of 3D multicellular cell spheroid models in anti-cancer drug discovery and development.

Cell-based assays are commonly used for high throughput screening (HTS) of anti-cancer drugs^1^. Screening assays are mostly performed on cells cultured on two-dimensional (2D) substrate, due to its simplicity and convenience. However, 2D cell culture has been postulated to contribute to poor translation of preclinical assays to the clinic, due to its highly artificial cellular environment that cannot reproduce the complexity and pathophysiology of tumor *in vivo*^2^,^3^.

3D multicellular spheroids as heterogeneous cellular aggregates have been considered to bridge the gap between 2D cell culture models and whole-animal models, since they closely reflect the *in vivo* pathophysiological situation in tumor tissues^4^,^5^,^6^. Compared to 2D monolayer cells, the gene expression in 3D spheroids is closer to clinical expression profile^7^,^8^,^9^. Furthermore, 3D multicellular spheroids reflect better the *in vivo* tumor environment in terms of phenotypic heterogeneity^10^,^11^, nutrient and oxygen gradients^12^,^13^, intervascular domains^14^, and micrometastases^15^. This technique was first applied in cancer search in 1970 by Sutherland *et al*.^16^. Since then, the 3D multicellular spheroid-based assays have been used for studying tumor growth^17^, migration^18^, invasion^19^, tumor angiogenesis^20^, and drug responsiveness^21^,^22^.

To generate tumor spheroids more effectively, several 3D culture methods have been developed, such as spontaneous aggregation^23^,^24^, rotary cell culture systems^23^,^25^, hanging drops^26^, liquid overlay on agar^27^,^28^,^29^, low binding plates^30^,^31^, and micropatterned plates^32^,^33^. Recently, a simple, standardized, and highly reproducible method has been established for 3D multicellular spheroid culture by combining round-bottom geometry with ultra-low attachment (ULA) surface chemistry, which is made readily in standard microtiter plate formats (e.g., 96-, 384-well)^18^,^29^,^34^. This technique has been validated to an increasing number of cancer cell types. However, 3D tumor spheroids of gastric cancer have not been developed via this technique.

Bufadienolides are C-24 steroids with a six membered lactone (α-pyrone) ring at the position of C-17β. Since the first bufadienolide scillaren A was isolated from Egyptian squill^35^, there have been more than 200 bufadienolides discovered via isolation from animals and plants or biotransformation^36^. These compounds have attracted great attention, since they show a wide range of bioactivities, such as renal sodium excretion, blood pressure stimulating^37^,^38^, immunoregulatory^39^,^40^, and anti-tumor activities towards diverse cancer cell lines, including hepatoma, lung carcinoma, pancreatic, gastro-intestinal and breast cancers^36^,^41^,^42^,^43^. These studies focus on their anti-tumor activities on 2D monolayer cells. Little is known about the activity of bufadienolides to inhibit the growth of cancer cells in 3D spheroid models.

Here, we aim to investigate anti-gastric cancer activity of natural bufadienolides using 3D spheroid-based functional assays.

## Results

### Establishment of 3D spheroid-based assays

**Figure 1:**
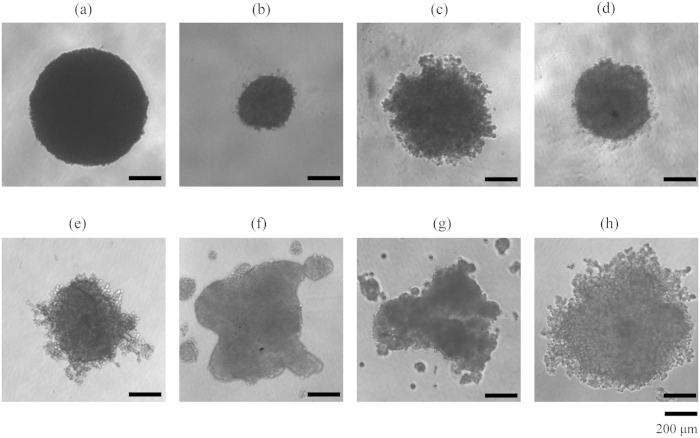
Morphology of multicellular spheroids formed in 96-well ULA round-bottomed plates after culturing for 4 days with the same cell density of 5000 cells per well, including HGC-27 (**a**), HT-29 (**b**), MDA-MB-231 (**c**), SUM-159 (**d**), A549 (**e**), Hep G2 (**f**), PLC/PRF/5 (**g**) and SK-HEP-1 (**h**). Scale bar: 200 μm.

To establish a 3D spheroid assay, we first examined the ability of multiple human cancer cell lines to form spheroid in the 96-well ULA round-bottomed plate. These cell lines included gastric cancer cell line HGC-27, colon carcinoma cell line HT-29, breast cancer cell lines MDA-MB-231 and SUM-159, lung cancer cell line A549 and hepatoma cell lines Hep G2, PLC/PRF/5 and SK-HEP-1. Results showed that different cell lines formed distinct types of spheroidal structures (Fig. 1). According to their spheroid morphology and the classification method in reference^18^,^44^, these spheroids were classified: tight spheroids (HGC-27, HT-29 and SUM-159), compact aggregates (MDA-MB-231 and A549), and loose aggregates (Hep G2, PLC/PRF/5 and SK-HEP-1). Interestingly, although HT-29 and SUM-159 cells formed tight spheroids, cells were dissociated from the spheroidal structure after treatment with anti-cancer drugs (data not shown), resulting in difficulty in measuring their diameters. In contrast, the gastric cancer cell line HGC-27 displayed a well-defined spheroid morphology in shape and was well packed, thus was chosen to develop 3D spheroid assays for compound profiling.

**Figure 2:**
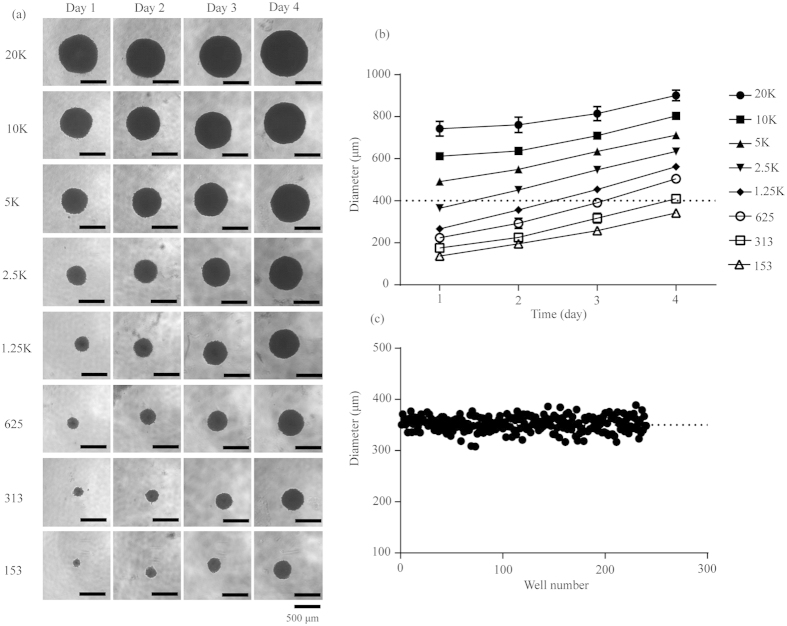
3D spheroid assay model of HGC-27. (**a**) Morphology of HGC-27 spheroids with different cell seeding densities on day 1-day 4. Scale bar: 500 μm. (**b**) The diameter of spheroids with different cell seeding densities as a function of culturing time in 96-well ULA round-bottomed plates. Data represents mean ± s.d. from 2 independent measurements, each in duplicate (n = 4). (**c**) Reproducibility of spheroids on day 4 across 4 plates, each plate containing 60 spheroids.

The physiological state of cells in spheroid depends on the spheroid size. A standard size of 370–400 μm after 4-day incubation was frequently selected for drug testing^29^. To obtain the optimal size of HGC-27 multicellular spheroids for screening, we further characterized its growth patterns as a function of initial seeding density. Results showed that cells with different seeding densities all formed tight and regular spheroids (Fig. 2a), but with different kinetics in increasing diameters over time (Fig. 2b). The size of spheroids was proportional to the initial cell numbers. With 300 cells per well, the spheroid diameter was approximately approaching 400 μm at day 4 with a low coefficient of variation (CV) (352.10 ± 14.62 μm, n = 240) in spheroid diameters, indicating great reproducibility in spheroid formation (Fig. 2c). Therefore, in the following studies, the cells with a seeding density of 300 cells per well cultured for 4 days in 96-well ULA round-bottomed plates were used for anti-cancer activity assays.

### Screening of anti-cancer activity of bufadienolides

**Figure 3:**
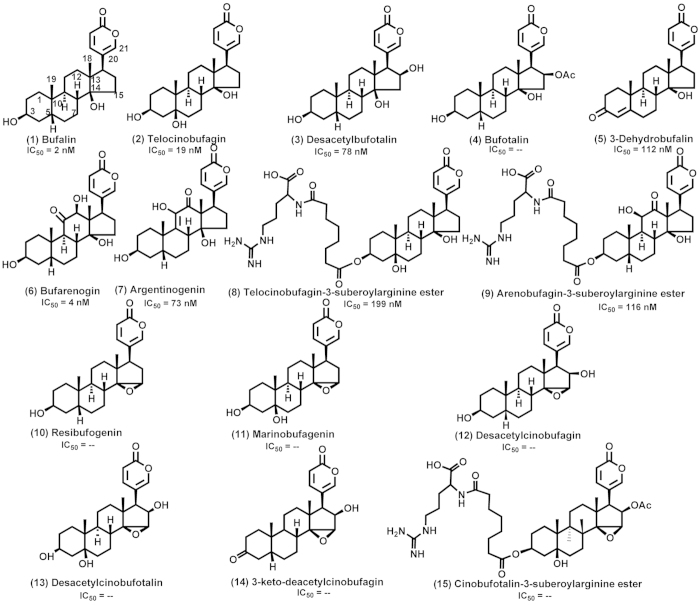
Chemical structures of 15 bufadienolides investigated in this work. IC_50_ was the values of bufadienolides on HGC-27 3D spheroids after treatment for 6 days and “–” means that the inhibitory active is weak.

**Figure 4:**
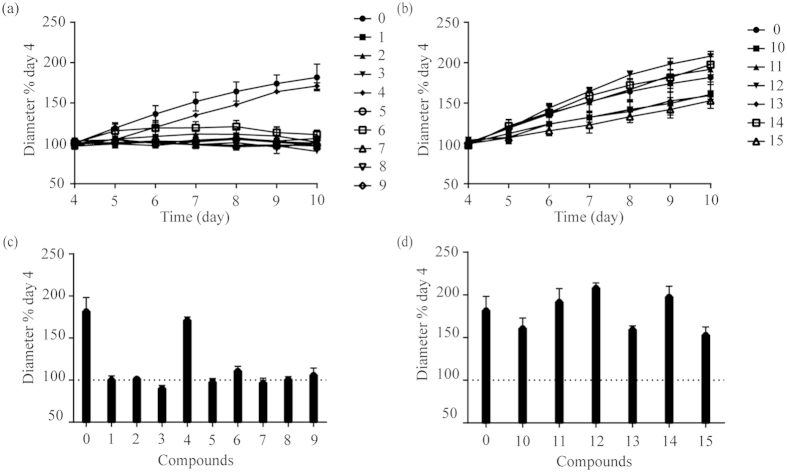
Screening of antitumor activity of 15 bufadienolides on HGC-27 cell spheroids. The growth kinetics of the 4-day-old spheroids treated with Group A (**a**) and Group B (**b**) compounds at the concentration of 400 nM. The spheroid size after treatment for 6 days with Group A (**c**) and Group B (**d**) compounds. “0” was the control. All data represents mean ± s.d. from 2 independent measurements, each in duplicate (n = 4).

We first profiled the anti-cancer activity of 15 bufadienolides (Fig. 3), each at 400 nM, using the HGC-27 3D spheroid growth assays. These bufadienolides belong to 14-hydroxy compounds (Group A) and 14, 15-epoxy compounds (Group B). Results showed that after treated with Group A compounds (except No. 4 bufotalin) the 4-day-old spheroids stopped growing as its size remained the same as the 4^th^ day for the next 6 days (Fig. 4a,c). On the other hand, compounds in Group B displayed overall weak inhibitory effects in spheroid growth (Fig. 4b,d). These results suggested that 14-hydroxy compounds had stronger anti-gastric cancer activities than 14, 15-epoxy compounds.

### Pharmacology of active bufadienolides

**Figure 5:**
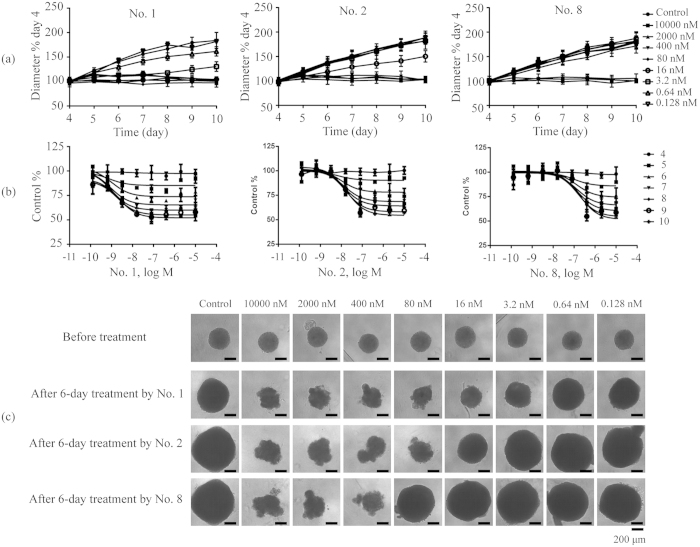
Bufadienolides concentration-dependent inhibition of HGC-27 cell spheroid growth. (**a**) The growth kinetics of the 4-day-old spheroids treated with No. 1, No. 2 and No. 8 compounds with different concentrations. (**b**) The concentration dependent curves of No. 1, No. 2 and No. 8 compounds after treatment for different days. (**c**) Morphology of HGC-27 spheroids after treatment for 6 days with No. 1, No. 2 and No. 8 compounds. Scale bar: 200 μm. Data represents mean ± s.d. from 2 independent measurements, each in duplicate (n = 4).

**Figure 6:**
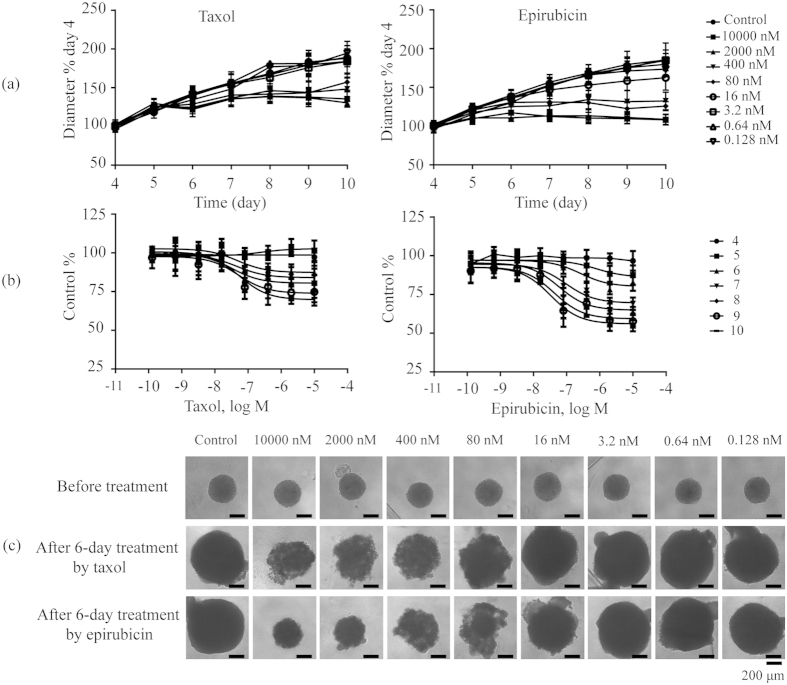
Taxol and epirubicin hydrochloride concentration-dependent inhibition of HGC-27 cell spheroid growth. (**a**) The growth kinetics of the 4-day-old spheroids treated with taxol and epirubicin with different concentrations. (**b**) The concentration dependent curves of taxol and epirubicin after treatment for different days. (**c**) Morphology of HGC-27 spheroids after treatment for 6 days with taxol and epirubicin. Scale bar: 200 μm. Data represents mean ± s.d. from 2 independent measurements, each in duplicate (n = 4).

We next examined the potency of active bufadienolides (compounds in Group A except No. 4) on HGC-27 3D multicellular spheroids. Results showed that all eight compounds tested completely stopped the spheroid growth when assayed at high concentration (Fig. 5). However, different compounds had different potency (Table 1), as well as different effective concentration range. Among them, bufalin (No. 1) had the widest effective concentration range, and telocinobufagin-3-suberoylarginine ester (No. 8) had the narrowest effective concentration range (Fig. 5a). For comparison, taxol and epirubicinboth had wide concentration range (Fig. 6a). These results suggest that active bufadienolides possessed the narrow effective concentration range to inhibit the growth of HGC-27 cells in spheroid.

Based on their inhibitory potency (Figs 3 and 5b, Table 1), bufalin (No. 1) was the most potent inhibitor. Compared to bufalin, introduction of 3-ketone (No. 5), 5-hydroxyl (No. 2) and 16-hydroxyl (No. 3) substituents reduced its potency. Introduction of 16-acetyl (No. 4) and 14,15-epoxide (No. 10) rendered compounds inactive. Introduction of 11-ketone and 12-hydroxyl (No. 6) made no significant difference in the degree of inhibitory activity. However, transformation of their positions (No. 7) would obviously lower the activity. Compounds No. 8 and No. 9 displayed weaker inhibitory activities than compounds No. 2 and No. 7, respectively, meaning that the large substituent at the C-3 position was unfavorable to the inhibitory activity.

**Figure 7:**
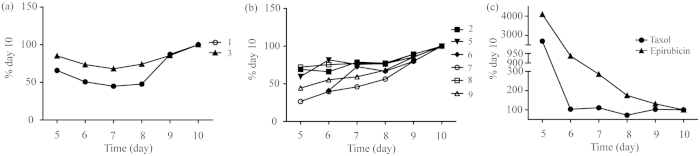
The change trend of IC_50_ values on HGC-27 spheroids after treatment for different time with No. 1 and No. 3 bufadienolides (**a**), No. 2 and No. 5–9 bufadienolides (**b**), and taxol and epirubicin hydrochloride (**c**).

Interestingly, different compounds also gave rise to distinct treatment duration dependent potency (Fig. 7). All bufadienolides exhibited decreasing potency as the treatment duration increases, opposite to both taxol and epirubicin, suggesting that these two clinical drugs exhibited longer lasting inhibitory effect on HGC-27 spheroids than bufadienolides. On the other hand, examining the morphology of HGC-27 spheroids after treatment for 6 days with these compounds (Fig. 5c) or drugs (Fig. 6c), revealed that similar to taxol and epirubicin, bufadienolides effectively penetrated the multiple layers of HGC-27 spheroids, as the outer-layer cells detached from spheroids due to exposure to compounds, possibly due to reduced cell-cell adhesion.

### Comparison anti-gastric cancer activity of bufadienolides in 3D multicellular spheroids with 2D monolayer cells

**Figure 8:**
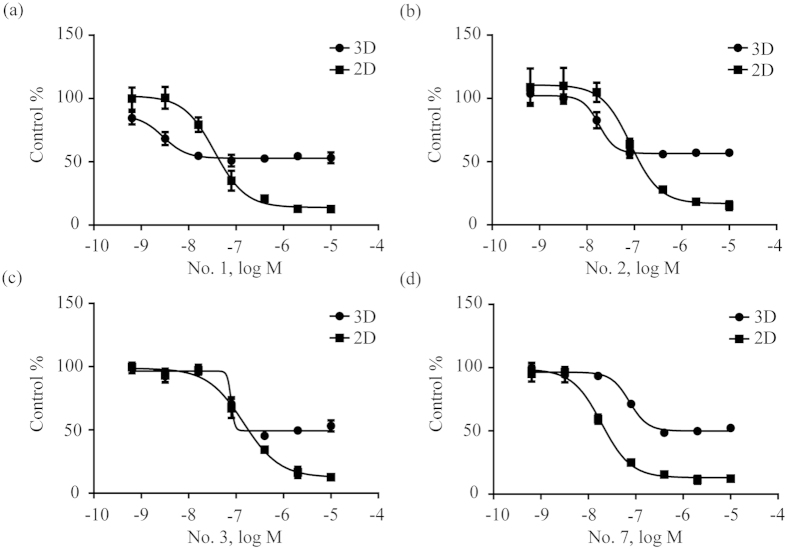
Cell viability in HGC-27 2D cell monolayer assays after 1-day treatment and spheroid diameter in HGC-27 3D multicellular spheroid assays after 6-day treatment by No. 1 (**a**), No. 2 (**b**), No. 3 (**c**) and No. 7 (**d**) bufadienolides. Data represents mean ± s.d. from 2 independent measurements, each in duplicate (n = 4).

Finally, we compared the pharmacology of bufadienolides in 3D tumor spheroids with 2D monolayer cells. Results showed that these compounds generally exhibited poorer efficacy, but different potency in 3D spheroid assay, compared to 2D cell monolayer assay (Fig. 8). Furthermore, the inhibitory potency of compounds in 3D spheroid-based assays decreased in the order: No. 1 > No. 6 > No. 2 > epirubicin > taxol > No. 7 > No. 3 > No. 5 > No. 9 > No. 8. However, the order of inhibitory potency in 2D cell monolayer assays was No. 6 > No. 7 > No. 1 > No. 2 > No. 3 > taxol > No. 9 > No. 5 > epirubicin > No. 8. Both 3D tumor spheroids and 2D cell monolayer assays showed three compounds No. 1, No. 6 and No. 2 all had better inhibitory potency than market drugs epirubicin and taxol. They may offer new therapeutic candidates to develop new treatment of gastric cancer. These results suggest that drug molecules may display different pharmacology in 3D multicellular spheroid models.

## Discussion

Bufadienolides display inhibitory activities towards various cancer cells^43^,^45^,^46^, which have great potential for becoming anti-cancer drug candidates. Current studies have focused on evaluation of the inhibitory activity of bufadienolides via 2D cell monolayer assays. However, owing to this highly artificial environment, 2D monolayer cells cannot mimic the pathophysiology of *in vivo* tumor, resulting in limitation for prediction of *in vivo* anti-cancer activities. 3D multicellular spheroids closely reflect the *in vivo* tumor characteristics, leading to better prediction power as a useful and effective technique for investigating anti-cancer activity of drugs^4^,^5^,^6^. Given that natural bufadienolides possess better inhibitory activity than derived bufadienolides^41^,^43^, 15 natural bufadienolides isolated from the skin of *Bufo bufo gargarizans* Cantor (toad skin) were screened and evaluated using 3D spheroid gastric cancer model.

Here, HGC-27 cells were found to spontaneously form the tightest spheroids in 96-well ULA round-bottomed microplate with high reproducibility. So 3D spheroid-based assays of HGC-27 cell line were used for compound screening and evaluation. These assays consisted of three steps: (1) spheroid formation via culturing cells at the optimal density for 4 days in 96-well ULA plate; (2) microscope imaging from the 4^th^ day to the 10^th^ day after addition of drugs or compounds on the 4^th^ day; (3) data process and analysis. According to procedures of 3D spheroid-based assays, 15 natural bufadienolides were screened, including 9 14-hydroxy compounds and 6 14, 15-epoxy compounds. Among them, 14-hydroxy compounds (except No. 4 bufotalin) had stronger anti-cancer activities than 14, 15-epoxy compounds, suggesting the importance of a hydroxy group in the C-14 position for the anti-cancer activities, which was consistent with result obtained on 2D cell monolayer assays and reported by Kamano *et al*.^41^. Interestingly, bufotalin (No. 4) belonged to 14-hydroxy compounds but displayed weak inhibitory activities. Overall, bufadienolide-induced growth inhibitory activity was validated in 3D spheroid-based assays.

Given that 3D spheroid-based assays can provide real-time quantitative kinetic analyses and detailed morphological investigations^18^,^29^,^34^, the growth kinetic curves, concentration-dependent curves and morphology of HGC-27 spheroids after treatment by active bufadienolides can be obtained. This discovery is appealing for investigation of pharmacology characteristics of drugs *in vivo*. According to their growth kinetic curves, active bufadienolides had relative narrow effective concentration range compared to taxol and epirubicin, which was unfavorable for their use in clinic due to their severe cardiotoxicity associated with specific inhibition of Na^+^/K^+^-ATPase^47^,^48^,^49^. As reported, bufalin was a potent inhibitor of Na^+^/K^+^-ATPase, with a cardiotoxicity range of 1 ~100 nM^50^. And its concentration range for inhibiting the growth of 3D multicellular spheroids was 0.64 ~16 nM. Thus, the narrow effective concentration range of bufadienolides for inhibiting the growth of 3D multicellular would be within the concentration range of cardiotoxicity, limiting their usage in clinic. Based on their inhibitory potency calculated by concentration-dependent curves, reasonable structure-activity relationships (SARs) were summarized and the change trend of IC_50_ values with time was acquired. It was found that large hydrophilic substituent at the C-3 position reduced the inhibitory potency, which could be attributed to the possibility that the hydrophilicity of the compounds may have an adverse effect on delivering compounds into the inner layer of 3D spheroids^51^. Compared to the long-lasting inhibitory effect on HGC-27 spheroids of the two clinical drugs, the inhibitory effect of active bufadienolides lasted for about three days and higher concentration of compounds was needed for longer time treatment, possibly due to drug-resistance^52^,^53^. According to spheroid morphology, active bufadienolides could effect cell-cell adhesion and effectively penetrate the multiple layers of HGC-27 spheroids, resulting in inhibiting their growth. These results provided important parameters for the development of their clinical usage.

Compared to 2D cell monolayer assays, compounds displayed different inhibitory activities in 3D spheroid-based assays. Firstly, all active bufadienolides showed weaker inhibitory efficacy in 3D spheroid-based assays, indicating that reduced compounds assessed to the center of multicellular spheroids. For instance, bufotalin (No. 4) had a higher inhibitory activity in 2D cell monolayer assay^41^, but almost lost its activities in 3D spheroid screening assays. Secondly, less bufadienolides were more active than clinical drug epirubicin in 3D spheroid-based assays. 7 bufadienolides had higher activities than epirubicin in 2D cell monolayer assays, whereas only 3 bufadienolides had higher activities than epirubicin in 3D spheroid-based assays. Moreover, their potency orders were very different. Thirdly, a more referable potency was obtained in 3D spheroid-based assays. The IC_50_ values of taxol and epirubicin on HGC-27 3D spheroids after treatment for 6 days were 0.062 ± 0.031 μM and 0.036 ± 0.015 μM, respectively. These values were within the range used in treatment^54^,^55^, confirming that 3D spheroid-based assays developed in this work could provide highly relevant screen for compounds. Therefore, the inhibitory activity of bufadienolides studied in this work would offer useful information on their related drug optimization and development.

## Methods

### Materials

Taxol and epirubicin hydrochloride were obtained from Melone biotechnology Co., Ltd. (Dalian, China). All bufadienolides were separated and prepared from toad skin in house using high performance liquid chromatography^56^,^57^, except marinobufagenin that was gifted from Prof. Ling Yang (Dalian Institute of Chemical Physics, Chinese Academy of Sciences, Dalian, China). The toad skin was collected from Shandong Province and authenticated by the Institute of Medication, Xiyuan hospital of China Academy of Traditional Chinese Medicine. All animal experimental procedures were carried out in accordance with the Guide for the Care and Use of Laboratory Animals promulgated by Ministry of Science and Technology of China, and were approved by the animal ethics committee of Xiyuan hospital of China Academy of Traditional Chinese Medicine. Their chemical structures are illustrated in Fig. 3. 96-well ULA round-bottomed plates and 96-well flat-bottomed plates were purchased from Corning Incorporated (Corning, NY, USA). *TransDect*^TM^ cell counting kit (CCK) was from Transgen biotechnology Co., Ltd. (Beijing, China). All compounds were dissolved in dimethyl sulfoxide (DMSO) and were stocked in 20 mM. All compounds were diluted freshly by phosphate buffer saline to the assayed concentrations.

### Cell culture

Human gastric cancer cell line HGC-27, colon carcinoma cell line HT-29, lung cancer cell line A549 and hepatoma cell lines Hep G2, PLC/PRF/5 and SK-HEP-1 were obtained from Cell Bank of Shanghai Institute of Cell Biology, Chinese Academy of Sciences. Human breast cancer cell lines MDA-MB-231 and SUM-159 were obtained from Second Affiliated Hospital of Dalian Medical University (Dalian, China). HGC-27, HT-29 and A549 cells were cultured using RPMI 1640, McCoy’s 5A and F12K medium, respectively. MDA-MB-231 and SUM-159 cells were cultured in Dulbecco’s Modified Eagle’s Medium (DMEM). Hep G2, PLC/PRF/5 and SK-HEP-1 cell lines were cultured in MEM medium. Except RPMI 1640 medium with 20% fetal bovine serum (FBS), all culture mediums contained 10% FBS. In addition, all culture mediums contained 50 μg/mL penicillin and 100 μg/mL streptomycin. All cell lines were cultured in a humidified 37 °C/5% CO_2_ incubator.

### Spheroid formation and screening of bufadienolides

Spheroids of different cell lines were formed in 96-well ULA round-bottomed plates by dispensing 200 μL cell suspensions with seeding density of 5000 cells per well. The morphology of multicellular spheroids were recorded using an inverted light microscope (Olympus CKX41, Olympus Co. Ltd., Tokyo, Japan) to choose proper cell lines.

Spheroids of HGC-27 cells were formed in 96-well ULA round-bottomed plates by dispensing 200 μL cell suspensions at different seeding densities 20000, 10000, 5000, 2500, 625, 313 and 156 cells per well. Optimal density for spheroid formation and screening drugs was assessed based on the size and shape of each seeding density from day 1 to day 4.

Cell suspension at the optimal density was seeded in 96-well ULA round-bottomed plates and 3D multicellular spheroids were spontaneously generated after 4 days culture. On day 4, 110 μL of the old media was exchanged by 100 μL of the fresh media in each well, and 10 μL of compound solution was then added. The size and shape of multicellular spheroids were recorded using an inverted light microscope from day 4 to day 10. During media changes, the pipet tip was held at about a 45° angle away from the center of the well to protect spheroids from disruption.

### Cell viability assay

The effects of all bufadienolides, taxol and epirubicin on HGC-27 cell proliferation in 2D monolayer culture were evaluated using CCK8 assay, each in four replicates. Specifically, HGC-27 cells were seeded in 96-well flat-bottomed plates at 1 × 10^4^ cells per well. After culture for 24 h, 100 μL of the old media was exchanged by 90 μL of the fresh media in each well, and 10 μL of compound solution was then added. The control was the wells that cells were treated by DMSO vehicle at a concentration equal to that in compound-treated cells. After treatment for 24 h, the media was removed from the wells and 100 μL of the media without FBS but containing 10% CCK8 was added in each well. After incubation at 37 °C for 2 h, the absorbance of each well was measured at 450 nm using a microplate reader (Bio-Rad, iMark, USA).

### Data analysis

The diameters of 3D multicellular spheroids were measured using NIH ImageJ (http://imagej.nih.gov/ij/). The suppression of the multicellular spheroid growth was normalized by the control. Data process and analysis were performed on Microsoft excel 2010 and GraphPad Prism 6.02 (GraphPad Sofware Inc., San Diego, CA, USA). All IC_50_ values described in this work were calculated based on two independent measurements, each in duplicate (n = 4).

## Additional Information

**How to cite this article**: Wang, J. *et al*. Anti-gastric cancer activity in three-dimensional tumor spheroids of bufadienolides. *Sci. Rep*. **6**, 24772; doi: 10.1038/srep24772 (2016).

## Figures and Tables

**Table 1:** The inhibitory activities of active bufadienolides on HGC-27 3D spheroids and 2D monolayer cells.

Compounds	3D						2D
	IC_50_^1^ (μM)	IC_50_^2^ (μM)	IC_50_^3^ (μM)	IC_50_^4^ (μM)	IC_50_^5^ (μM)	IC_50_^6^ (μM)	IC_50_(μM)
No.1	0.002 ± 0.001	0.001 ± 0.0006	0.001 ± 0.0005	0.001 ± 0.0004	0.002 ± 0.0008	0.002 ± 0.0008	0.034 ± 0.005
No.2	0.013 ± 0.011	0.012 ± 0.007	0.015 ± 0.006	0.014 ± 0.005	0.017 ± 0.006	0.019 ± 0.005	0.087 ± 0.017
No.3	0.067 ± 0.044	0.058 ± 0.024	0.053 ± 0.02	0.058 ± 0.019	0.067 ± 0.019	0.078 ± 0.026	0.152 ± 0.019
No.5	0.067 ± 0.046	0.091 ± 0.045	0.085 ± 0.04	0.086 ± 0.034	0.096 ± 0.036	0.112 ± 0.041	0.285 ± 0.057
No.6	–	0.002 ± 0.001	0.003 ± 0.002	0.003 ± 0.001	0.003 ± 0.001	0.004 ± 0.001	0.014 ± 0.002
No.7	0.019 ± 0.013	0.029 ± 0.007	0.034 ± 0.01	0.041 ± 0.014	0.058 ± 0.015	0.073 ± 0.018	0.018 ± 0.003
No.8	0.144 ± 0.104	0.15 ± 0.082	0.154 ± 0.076	0.152 ± 0.065	0.178 ± 0.078	0.199 ± 0.082	1.044 ± 0.274
No.9	0.051 ± 0.043	0.064 ± 0.038	0.069 ± 0.033	0.079 ± 0.031	0.099 ± 0.038	0.116 ± 0.04	0.279 ± 0.062
Taxol	1.683 ± 1.667	0.065 ± 0.044	0.069 ± 0.052	0.045 ± 0.031	0.064 ± 0.041	0.062 ± 0.031	0.275 ± 0.059
Epirubicin	1.488 ± 1.27	0.34 ± 0.217	0.104 ± 0.045	0.064 ± 0.028	0.048 ± 0.022	0.036 ± 0.015	1.041 ± 0.146

Notes: (1) IC_50_^1^, IC_50_^2^, IC_50_^3^, IC_50_^4^, IC_50_^5^ and IC_50_^6^ are the IC_50_ values of bufadienolides on HGC-27 3D spheroids after treatment for 1, 2, 3, 4, 5 and 6 days, respectively. (2) “–” means that the inhibitory active is weak.

## References

[b1] Sharma S. V., Haber D. A. & Settleman J. Cell line-based platforms to evaluate the therapeutic efficacy of candidate anticancer agents. Nat. Rev. Cancer 10, 241–253 (2010). 20300105 10.1038/nrc2820

[b2] Abbott A. Cell culture: Biology’s new dimension. Nature 424, 870–872 (2003). 12931155 10.1038/424870a

[b3] Hutchinson L. & Kirk R. High drug attrition rates-where are we going wrong? Nat. Rev. Clin. Oncol. 8, 189–190 (2011). 21448176 10.1038/nrclinonc.2011.34

[b4] Muellerklieser W. Multicellular spheroids-a review on cellular aggregates in cancer-research. J. Cancer Res. Clin. Oncol. 113, 101–122 (1987). 3549738 10.1007/BF00391431 PMC12248366

[b5] Pampaloni F., Reynaud E. G. & Stelzer E. H. K. The third dimension bridges the gap between cell culture and live tissue. Nat. Rev. Mol. Cell Biol. 8, 839–845 (2007). 17684528 10.1038/nrm2236

[b6] Hirschhaeuser F. . Multicellular tumor spheroids: An underestimated tool is catching up again. J. Biotechnol. 148, 3–15 (2010). 20097238 10.1016/j.jbiotec.2010.01.012

[b7] Chang T. T. & Hughes-Fulford M. Monolayer and Spheroid Culture of Human Liver Hepatocellular Carcinoma Cell Line Cells Demonstrate Distinct Global Gene Expression Patterns and Functional Phenotypes. Tissue Eng. Part A 15, 559–567 (2009). 18724832 10.1089/ten.tea.2007.0434 PMC6468949

[b8] De Witt Hamer P. C. . The genomic profile of human malignant glioma is altered early in primary cell culture and preserved in spheroids. Oncogene 27, 2091–2096 (2008). 17934519 10.1038/sj.onc.1210850

[b9] Ernst A. . Genomic and Expression Profiling of Glioblastoma Stem Cell-Like Spheroid Cultures Identifies Novel Tumor-Relevant Genes Associated with Survival. Clin. Cancer Res. 15, 6541–6550 (2009). 19861460 10.1158/1078-0432.CCR-09-0695

[b10] Busse A. . Characterization of small spheres derived from various solid tumor cell lines: are they suitable targets for T cells? Clin. Exp. Metastasis 30, 781–791 (2013). 23519726 10.1007/s10585-013-9578-5

[b11] Lawrenson K. . Modelling genetic and clinical heterogeneity in epithelial ovarian cancers. Carcinogenesis 32, 1540–1549 (2011). 21859834 10.1093/carcin/bgr140

[b12] Ghosh S. . Three-dimensional culture of melanoma cells profoundly affects gene expression profile: A high density oligonucleotide array study. J. Cell. Physiol. 204, 522–531 (2005). 15744745 10.1002/jcp.20320

[b13] Chiba M., Yokoyama C., Okada M. & Hisatomi H. Mitochondrial DNA reduced by hypoxic conditions in three-dimensional (3D) spheroid cell cultures. Tumor Biol. 35, 12689–12693 (2014). 10.1007/s13277-014-2593-6 25217322

[b14] Friedrich J., Ebner R. & Kunz-Schughart L. A. Experimental anti-tumor therapy in 3-D: spheroids–old hat or new challenge? Int. J. Radiat. Biol. 83, 849–871 (2007). 18058370 10.1080/09553000701727531

[b15] Sutherland R. M. Cell and environment interactions in tumor microregions-the multicell spheroid model. Science 240, 177–184 (1988). 2451290 10.1126/science.2451290

[b16] Sutherland R. M., McCredie J. A. & Inch W. R. Growth of multicell spheroids in tissue culture as a model of nodular carcinomas. J. Natl. Cancer Inst. 46, 113–120 (1971). 5101993

[b17] Chitcholtan K., Asselin E., Parent S., Sykes P. H. & Evans J. J. Differences in growth properties of endometrial cancer in three dimensional (3D) culture and 2D cell monolayer. Exp. Cell Res. 319, 75–87 (2013). 23022396 10.1016/j.yexcr.2012.09.012

[b18] Vinci M. . Advances in establishment and analysis of three-dimensional tumor spheroid-based functional assays for target validation and drug evaluation. BMC Biol. 10, 1–20 (2012). 22439642 10.1186/1741-7007-10-29 PMC3349530

[b19] Harma V. . A Comprehensive Panel of Three-Dimensional Models for Studies of Prostate Cancer Growth, Invasion and Drug Responses. PLos One 5, 1–17 (2010). 10.1371/journal.pone.0010431 PMC2862707 20454659

[b20] Ghosh S. . Use of multicellular tumor spheroids to dissect endothelial cell-tumor cell interactions: A role for T-cadherin in tumor angiogenesis. FEBS Lett. 581, 4523–4528 (2007). 17765896 10.1016/j.febslet.2007.08.038

[b21] Pickl M. & Ries C. H. Comparison of 3D and 2D tumor models reveals enhanced HER2 activation in 3D associated with an increased response to trastuzumab. Oncogene 28, 461–468 (2009). 18978815 10.1038/onc.2008.394

[b22] Luca A. C. . Impact of the 3D Microenvironment on Phenotype, Gene Expression, and EGFR Inhibition of Colorectal Cancer Cell Lines. PLos One 8, 1–11 (2013). 10.1371/journal.pone.0059689 PMC3608563 23555746

[b23] Deisboeck T. S. . Pattern of self-organization in tumour systems: complex growth dynamics in a novel brain tumour spheroid model. Cell Prolif. 34, 115–134 (2001). 11348426 10.1046/j.1365-2184.2001.00202.x PMC6495396

[b24] Singh S. K. . Identification of a cancer stem cell in human brain tumors. Cancer Res. 63, 5821–5828 (2003). 14522905

[b25] Unsworth B. R. & Lelkes P. I. Growing tissues in microgravity. Nat. Med. 4, 901–907 (1998). 9701241 10.1038/nm0898-901

[b26] Del Duca D., Werbowetski T. & Del Maestro R. F. Spheroid preparation from hanging drops: characterization of a model of brain tumor invasion. J. Neuro-Oncol. 67, 295–303 (2004). 10.1023/b:neon.0000024220.07063.70 15164985

[b27] Weiswald L.-B. . *In situ* protein expression in tumour spheres: development of an immunostaining protocol for confocal microscopy. Bmc Cancer 10, 1–11 (2010). 20307308 10.1186/1471-2407-10-106 PMC2851689

[b28] Li Q. . 3D Models of Epithelial-Mesenchymal Transition in Breast Cancer Metastasis: High-Throughput Screening Assay Development, Validation, and Pilot Screen. J. Biomol. Screen 16, 141–154 (2011). 21297102 10.1177/1087057110392995

[b29] Friedrich J., Seidel C., Ebner R. & Kunz-Schughart L. A. Spheroid-based drug screen: considerations and practical approach. Nat. Protoc. 4, 309–324 (2009). 19214182 10.1038/nprot.2008.226

[b30] Wright M. H. . Brca1 breast tumors contain distinct CD44(+)/CD24(−) and CD133(+) cells with cancer stem cell characteristics. Breast Cancer Res. 10, 1–16 (2008). 10.1186/bcr1855 PMC2374965 18241344

[b31] Takaishi S. . Identification of Gastric Cancer Stem Cells Using the Cell Surface Marker CD44. Stem Cells 27, 1006–1020 (2009). 19415765 10.1002/stem.30 PMC2746367

[b32] Matsuda Y. . Morphological and cytoskeletal changes of pancreatic cancer cells in three-dimensional spheroidal culture. Med. Mol. Morphol. 43, 211–217 (2010). 21267697 10.1007/s00795-010-0497-0

[b33] Hardelauf H. . Microarrays for the scalable production of metabolically relevant tumour spheroids: a tool for modulating chemosensitivity traits. Lab Chip 11, 419–428 (2011). 21079873 10.1039/c0lc00089b

[b34] Febles N. K., Ferrie A. M. & Fang Y. Label-Free Single Cell Kinetics of the Invasion of Spheroidal Colon Cancer Cells through 3D Matrigel. Anal. Chem. 86, 8842–8849 (2014). 25118958 10.1021/ac502269v

[b35] Stoll A., Suter E., Kreis W., Bussemaker B. & Hofmann A. Heart-activating substances of squill. scillaren, I. Heart glucosides. Helv. Chim. Acta 16, 703–733 (1933).

[b36] Gao H. M., Popescu R., Kopp B. & Wang Z. M. Bufadienolides and their antitumor activity. Nat. Prod. Rep. 28, 953–969 (2011). 21416078 10.1039/c0np00032a

[b37] Doris P. A. & Bagrov A. Y. Endogenous sodium pump inhibitors and blood pressure regulation: An update on recent progress. Proc. Soc. Exp. Biol. Med. 218, 156–167 (1998). 9648933 10.3181/00379727-218-44283

[b38] Dmitrieva R. I. & Doris P. A. Cardiotonic steroids: Potential endogenous sodium pump ligands with diverse function. Exp. Biol. Med. 227, 561–569 (2002). 10.1177/153537020222700803 12192097

[b39] Terness P., Navolan D., Dufter C., Kopp B. & Opelz G. Exquisitely small amounts of nonglucocoriticoid natural steroids suppress the human allogeneic T-cell response. Transplant. Proc. 33, 547–548 (2001). 11266951 10.1016/s0041-1345(00)02135-7

[b40] Terness P., Navolan D., Dufter C., Kopp B. & Opelz G. The T-cell suppressive effect of bufadienolides: structural requirements for their immunoregulatory activity. Int. Immunopharmacol. 1, 119–134 (2001). 11367509 10.1016/s0162-3109(00)00264-2

[b41] Kamano Y. . QSAR evaluation of the Ch’an Su and related bufadienolides against the colchicine-resistant primary liver carcinoma cell line PLC/PRF/51. J. Med. Chem. 45, 5440–5447 (2002). 12459012 10.1021/jm0202066

[b42] Yin P.-H. . Anti-tumor Activity and Apoptosis-regulation Mechanisms of Bufalin in Various Cancers: New Hope for Cancer Patients. Asian Pac. J. Cancer Prev. 13, 5339–5343 (2012). 23317181 10.7314/apjcp.2012.13.11.5339

[b43] Moreno Y., Banuls L. . Structure-activity relationship analysis of bufadienolide-induced *in vitro* growth inhibitory effects on mouse and human cancer cells. J. Nat. Prod. 76, 1078–1084 (2013). 23706005 10.1021/np400034d

[b44] Ivascu A. & Kubbies M. Diversity of cell-mediated adhesions in breast cancer spheroids. Int. J. Oncol. 31, 1403–1413 (2007). 17982667

[b45] Dong Y. . Bufadienolide compounds sensitize human breast cancer cells to TRAIL-induced apoptosis via inhibition of STAT3/Mcl-1 pathway. Apoptosis 16, 394–403 (2011). 21259053 10.1007/s10495-011-0573-5

[b46] Meng Z. . Pilot study of huachansu in patients with hepatocellular carcinoma, nonsmall‐cell lung cancer, or pancreatic cancer. Cancer 115, 5309–5318 (2009). 19701908 10.1002/cncr.24602 PMC2856335

[b47] Jiang J. . Toxic effect of Chansu on cardiac electrophysiology in guinea-pigs. Chinese J. Pharmacol. Toxicol. 25, 307–309 (2011).

[b48] Ojiri Y., Noguchi K. & Sakanashi M. Effects of a senso (toad venom) containing drug on systemic hemodynamics, cardiac-function and myocardial oxygen-consumption in anesthetized dogs. Am. J. Chin. Med. 19, 17–31 (1991). 1897488 10.1142/S0192415X91000041

[b49] Prassas I. & Diamandis E. P. Novel therapeutic applications of cardiac glycosides. Nat. Rev. Drug Discov. 7, 926–935 (2008). 18948999 10.1038/nrd2682

[b50] Tian H.-Y. . C-23 Steroids from the Venom of Bufo bufo gargarizans. J. Nat. Prod. 76, 1842–1847 (2013). 24050254 10.1021/np400174f

[b51] Mehta G., Hsiao A. Y., Ingram M., Luker G. D. & Takayama S. Opportunities and challenges for use of tumor spheroids as models to test drug delivery and efficacy. J. Control. Release 164, 192–204 (2012). 22613880 10.1016/j.jconrel.2012.04.045 PMC3436947

[b52] Gottesman M. M. Mechanisms of cancer drug resistance. Annu. Rev. Med. 53, 615–627 (2002). 11818492 10.1146/annurev.med.53.082901.103929

[b53] Holohan C., Van Schaeybroeck S., Longley D. B. & Johnston P. G. Cancer drug resistance: an evolving paradigm. Nat. Rev. Cancer 13, 714–726 (2013). 24060863 10.1038/nrc3599

[b54] Wilson W. H. . Paclitaxel in doxorubicin-refractory or mitoxantrone-refractory breast cancer: a phase I/II trial of 96-hour infusion. J. Clin. Oncol. 12, 1621–1629 (1994). 7913721 10.1200/JCO.1994.12.8.1621

[b55] Bastholt L. . Dose-response relationship of epirubicin in the treatment of postmenopausal patients with metastatic breast cancer: a randomized study of epirubicin at four different dose levels performed by the Danish Breast Cancer Cooperative Group. J. Clin. Oncol. 14, 1146–1155 (1996). 8648369 10.1200/JCO.1996.14.4.1146

[b56] Li X. L. . Efficient purification of active bufadienolides by a class separation method based on hydrophilic solid-phase extraction and reversed-phase high performance liquid chromatography. J. Pharm. Biomed. Anal. 97, 54–64 (2014). 24814996 10.1016/j.jpba.2014.04.015

[b57] Li X. L. . Purification of bufadienolides from the skin of Bufo bufo gargarizans Cantor with positively charged C18 column. J. Pharm. Biomed. Anal. 92, 105–113 (2014). 24503198 10.1016/j.jpba.2014.01.002

